# The upregulation of peripheral CD3^-^CD56^+^CD16^+^ natural killer cells correlates with Th1/Th2 imbalance in asthma patients during acute upper respiratory viral infections

**DOI:** 10.1186/s12865-023-00575-y

**Published:** 2023-10-21

**Authors:** Meixuan Liu, Yunxuan Zhang, Yunqian Hu, Zhongliang Guo, Lin Dong

**Affiliations:** 1 Department of Respiratory Medicine, Shanghai East Hospital, School of Medicine, Tongji University, 200123 Shanghai, China; 2 Department of Pharmacy, Huadong Hospital, Fudan University, 200120 Shanghai, China; 3 Department of Thoracic Surgery, Shanghai East Hospital, School of Medicine, Tongji University, 200123 Shanghai, China

**Keywords:** NK cell, Th1/Th2 immunity, Viral Infection, Cytokines, Asthma

## Abstract

**Purpose:**

The aim of this study is to clarify the changes of peripheral CD3^−^CD56^+^CD16^+^ NK cells and their correlation with Th1/Th2 immunity profiles in asthma during the phase of acute upper respiratory viral infections (AURVIs).

**Methods:**

Peripheral venous blood and induced sputum samples were collected from 56 mild asthma patients, 49 asthma patients with AURVIs and 50 healthy subjects. Peripheral CD3^−^CD56^+^CD16^+^ NK cells were monitored by flow cytometry during the course of acute viral infections. Meanwhile, the induced sputum Th2 cytokines IL-4 and IL-5, and Th1 cytokine IFN-γ were also detected by ELISA assay.

**Results:**

The asthmatics had lower levels of peripheral CD3^−^CD56^+^CD16^+^ NK cells populations as well as higher induced sputum cytokines (IL-4, IL-5 and IFN-γ) compared to healthy controls at baseline. Upon upper respiratory viral infections, peripheral CD3^−^CD56^+^CD16^+^ NK cells numbers in asthma patients sharply elevated on day 3 and slowly decreased by day 14, in accordance with induced sputum IFN-γ changes. IL-4 and IL-5 levels spiked much later (day 8) and lasted until day 14. Compared with asthma alone group, the IFN-γ/IL-4 and IFN-γ/IL-5 ratios of the asthma patients with AURVIs on day 1 were higher and peaked on day 3. The changes of peripheral CD3^−^CD56^+^CD16^+^ NK cells proportions positively correlated with the IFN-γ/IL-4 and IFN-γ/IL-5 ratios on day 1 to day 3 in asthma subsequent to upper respiratory viral infections.

**Conclusions:**

Our findings showed an imbalanced Th1/Th2 immunity in airways of asthma with acute upper respiratory viral infections. Upregulated peripheral CD3^−^CD56^+^CD16^+^ NK cells play a crucial role in biased Th1 immunity of airways in asthma during the acute phase of viral infections. The anti-viral Th1 immunity by targeting NK cells may be a possible therapeutic option for virus-induced asthma exacerbation.

## Introduction

Asthma is one of the most common chronic respiratory illnesses characterized by airway inflammation, airway hyperresponsiveness and tissue remodeling that eventually lead to the clinical manifestations including cough, wheezing, breathless and reversible airflow limitation [1, 2]. The prevalent rate of this disease has rapidly increased over recent decades and affects about 334 million people in the world with a serious healthcare expense [3, 4]. Respiratory viral infections are recognized as a main cause of asthma and asthma exacerbation [5, 6]. Previous studies reveal that acute upper respiratory viral infections (AURVIs) in childhood are a high risk factor for subsequently developing asthma. Besides, respiratory syncytial virus (RSV)-induced acute bronchiolitis weakens the effect of corticosteroid for controlling asthma-related symptoms [7]. As a heterogeneous disease, asthma pathogenesis involves multiple cell types and inflammatory mediators performing innate and adaptive immunity. However, the inflammatory and immune responses in the pathophysiology of asthma during respiratory viral infections have not been extensively understood.

Natural killer (NK) cells are a subset of innate lymphocytes and generally participate in pathogen infection and tumor immunology with the cytotoxic functions. In particular, emerging data have confirmed the defensive role of NK cells against bacterial and viral insults through the secretion of cytokines and chemokines [8, 9]. Moreover, NK cells may regulate the immune response by interacting with other cells such as T lymphocytes in antiviral defense [10, 11]. Given T helper 2 (Th2) cells -mediated immune response via producing type 2 cytokines (IL-4, IL-5 and IL-13) plays a key role for the hallmark features of asthma, we speculate that NK cells exert important influence on the initiation and maintenance of immunity in asthma in response to virus infections. CD3^−^CD56^+^CD16^+^ NK cells subpopulations are activated forms of NK cells under inflammatory microenvironments [12, 13]. However, the impact of CD3^−^CD56^+^CD16^+^ NK cells on the balance of Th1/Th2 immunity in asthma is poorly described. In addition, little information is known regarding the profiles of airway inflammation during the phase of AURVIs. In human, peripheral blood CD56 + NK cells have been shown to be primed during acute respiratory viral infections [14]. More importantly, the vast majority of NK cells in the human lung are circulating between the organ and peripheral blood [15]. Therefore, the evaluation of circulating cell function and activation during acute infection is a simple and effective method. For another, induced sputum cytokines may directly reflect airway inflammation in asthmatic patients. Based on this concept, we collected peripheral blood and induced sputum samples from mild asthmatic patients with AURVIs. The frequencies of CD3^−^CD56^+^CD16^+^ NK cells and their interactions with the Th1/Th2 cytokines following respiratory virus infections were further evaluated. The purpose of this study is to explore the changes of CD3^−^CD56^+^CD16^+^ NK cells and their correlations with airway Th1/Th2 immune patterns in asthma subsequent to AURVIs.

## Results

### Baseline clinical characteristics

The baseline characteristics of all subjects were summarized in Table 1. There were no significant differences in terms of age, gender and BMI among the three groups. Among the asthma patients with AURVIs, 21 had RSV infections, 11 had rhinovirus infections, 4 had flu virus infections, 5 had adenovirus infections and 8 had parainfluenza virus infections. Compared with healthy subjects, these two asthmatic groups had higher levels of IgE, FeNO, sputum cell counts and atopy. Worsen lung functions were found in the two asthmatic groups. The rates of FEV1/predicted and FEV1/FVC were significantly lower in asthma patients with AURVIs than those in asthma alone group. Besides, relative to asthma alone group, the asthma patients with AURVIs had poor symptom controls analyzed by ACT and ACQ scores. The two asthma groups had higher sputum EOS counts than healthy controls, but no significant difference was found in blood EOS counts among the three groups.

**Table 1:** Baseline Clinical Characteristics of the three studied groups

	Asthma (n = 56)	Asthma with AURVIs (n = 49)	Heathy controls (n = 50)	*p* value
Age(years)	42.55 ± 8.84 (26 ~ 58)	40.76 ± 10.82 (22 ~ 58)	40.80 ± 9.81 (25 ~ 55)	0.558
Gender(M:F)	27:29	23:26	27:23	0.686
BMI(kg/m2)	21.69 ± 2.45	22.06 ± 1.85	21.59 ± 1.97	0.507
Virus strain				
Respiratory syncytial virus	/	21	/	/
Human rhinovirus	/	11	/	/
Influenza	/	4	/	/
adenovirus	/	5	/	/
Parainfluenza virus	/	8	/	/
IgE (IU/mL)	85.49(23 ~ 465)^a^	89.04(21 ~ 420)^a^	58.79(22 ~ 223)	0.001
Atopy (yes/no)	21/35 ^a^	19/30 ^a^	7/43	0.009
FeNO (ppb)	37.83 ± 17.61 ^a^	42.10 ± 17.36 ^a^	21.84 ± 6.49	< 0.001
FEV1/predicted (%)	86.69 ± 12.73 ^a^	79.94 ± 13.08 ^a^	100.05 ± 10.84	< 0.001
FEV1/FVC (%)	83.48 ± 8.04^a^	77.01 ± 10.72 ^a^	89.37 ± 9.01	< 0.001
ACT scores	21.39 ± 2.02	19.00 ± 2.52 ^b^	/	< 0.001
ACQ scores	1.18 ± 0.53	1.97 ± 0.74 ^b^	/	< 0.001
Haematology				
Leukocyte(10^9^/L)	6.43 ± 1.89	6.15 ± 1.81	5.89 ± 1.63	0.305
Eosinophil(%)	2.60 ± 1.11	3.02 ± 1.51	2.56 ± 1.38	0.163
Neutrophil (%)	57.31 ± 13.21	61.07 ± 12.96	56.35 ± 9.79	0.125
Eosinophil in induced sputum(%)	7.71(0.31 ~ 14.85) ^a^	6.85(0.21 ~ 16.32) ^a^	2.35(0 ~ 8.14)	< 0.001
Neutrophil in induced sputum(%)	64.78(43.68 ~ 93.32) ^a^	65.33(40.69 ~ 91.25) ^a^	47.18(15.52 ~ 76.95)	< 0.001

^a^*p* < 0.05 vs. healthy controls, ^b^*p* < 0.05 vs. asthma group

### Time trends of Th1 and Th2 cytokines in induced sputum

**Fig. 1:**
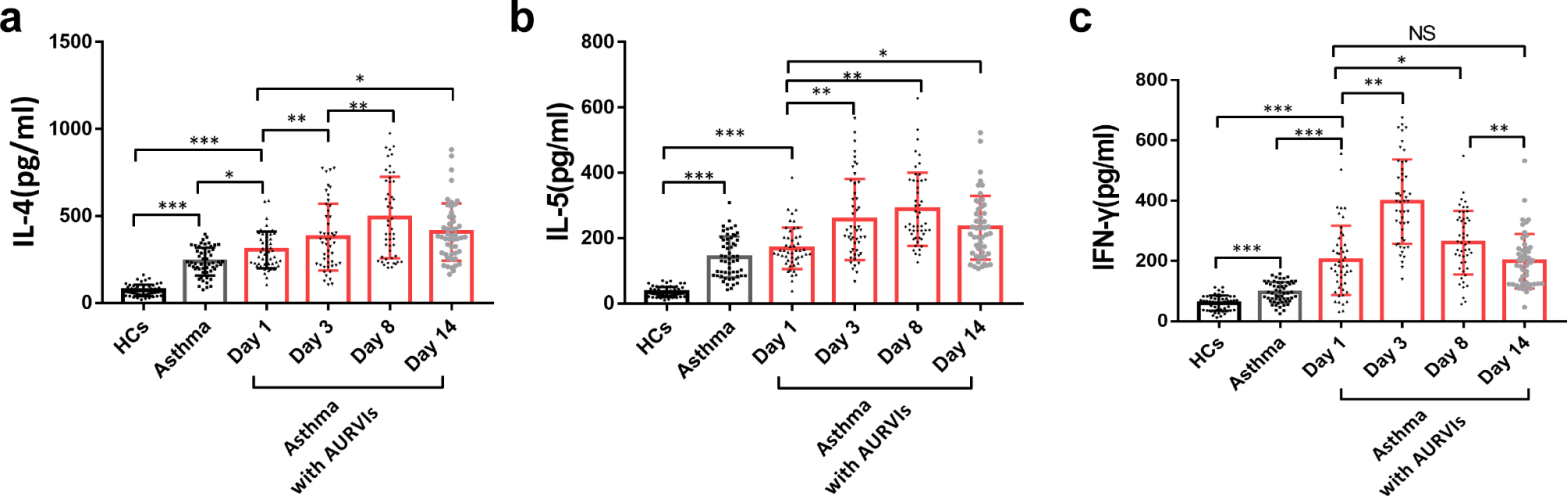
Comparisons of inflammatory cytokines (**a**) IL-4, (**b**) IL-5 and (**c**) IL-13 in induced sputum among healthy controls (N = 50), asthmatic subjects (N = 56), and asthma patients with (N = 49) on day 1, day 3,day 8 and day 14. *p < 0.05, ** p < 0.01, *** p < 0.001

To investigate the pattern of Th1/Th2 immunity in the airways of asthmatics upon viral infections, the type 2- ( IL-4 and IL-5) and type 1-( IFN-γ) inflammatory markers were measured in induced sputum from the individuals. Initially, compared to the healthy controls, the two asthma groups were found to have higher baseline levels of Th2 cytokines IL-4 (238.13 ± 88.45 ng/ml versus 72.84 ± 33.27 ng/ml, p < 0.001; 305.01 ± 105.59 ng/ml versus 72.84 ± 33.27 ng/ml, p < 0.001) and IL-5 (142.50 ± 63.05 ng/ml versus 35.66 ± 15.67 ng/ml, p < 0.001; 169.16 ± 63.67 ng/ml versus 35.66 ± 15.67 ng/ml, p < 0.001). Besides, the initial IL-4 levels in the asthma with AURVIs group were greatly upregulated compared with the asthma alone group (305.01 ± 105.59 ng/ml versus 238.13 ± 88.45 ng/ml, p = 0.023). Following viral infections, concentrations of sputum IL-4 and IL-5 in the asthma subjects significantly increased up to day 3, and afterwards peaked on day 8 during the observation period. On day 14, it was found that IL-4 and IL-5 levels were slightly declined, yet were higher than the values on day 1 (Fig. 1a and b). Regarding IFN-γ, the baseline values in the asthma alone group were significantly elevated relative to the controls (95.55 ± 33.92 ng/ml versus 59.68 ± 25.25 ng/ml, p < 0.001). Importantly, asthmatic patients with AURVIs on day 1 had higher IFN-γ levels than asthma alone group (201.94 ± 114.81 ng/ml versus 59.68 ± 25.25 ng/ml, p < 0.001). For the patients with both asthma and AURVIs, sputum levels of IFN-γ were sharply increased from day 1 to day 3 (201.94 ± 114.81 ng/ml versus 396.90 ± 139.95 ng/ml, p < 0.001), and then decreased to day 8. It was also found that the IFN-γ levels decreased to the lowest values on day 14 which were similar to the baseline levels on day 1 (199.08 ± 90.50 ng/ml versus 201.94 ± 114.81 ng/ml, p = 0.88) (Fig. 1c).

### Changes of CD3^−^CD56^+^CD16^+^ NK cells in Asthma patients during respiratory virus Infections

**Fig. 2:**
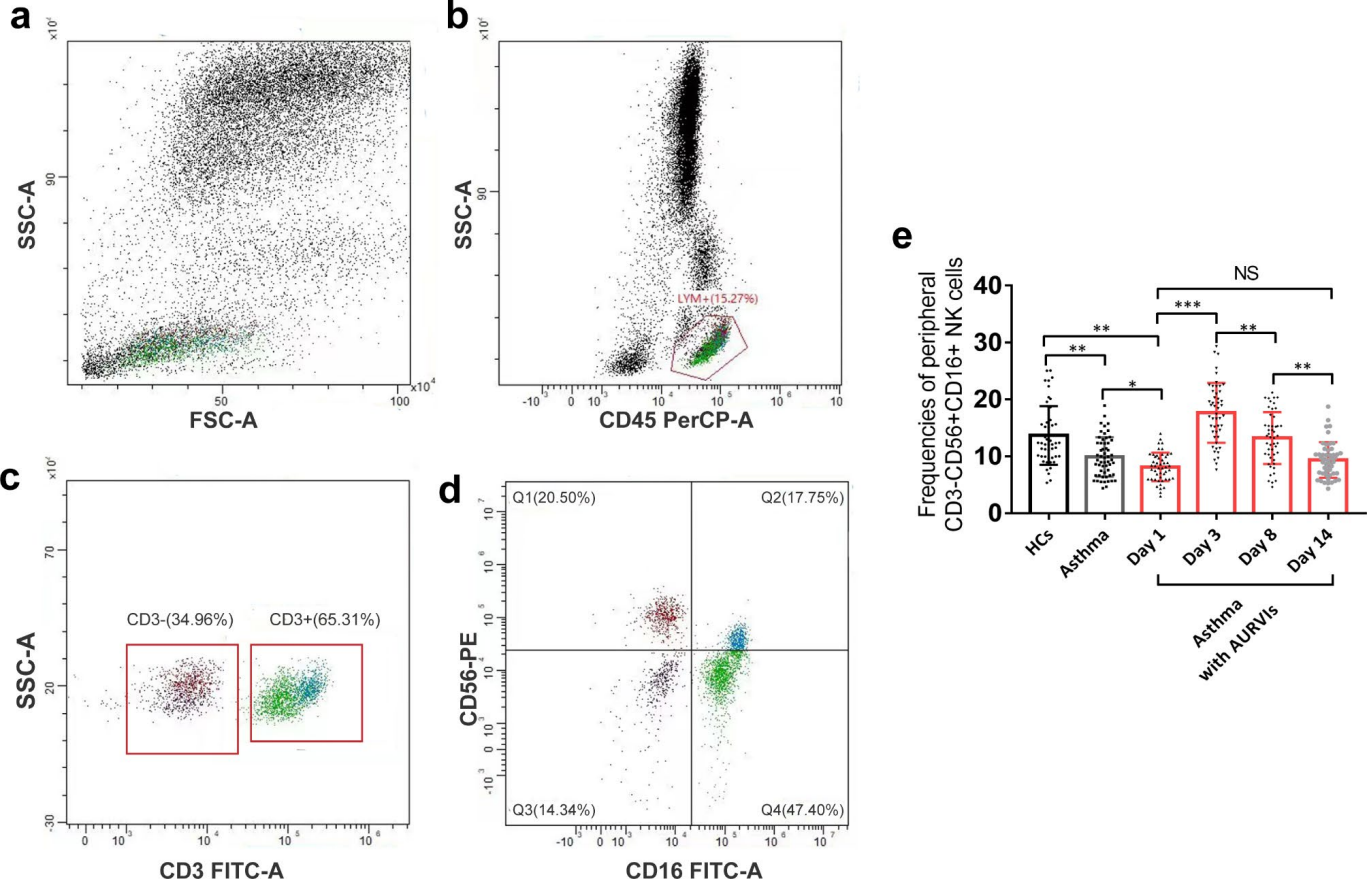
The proportions of CD3^−^CD56^+^CD16^+^ NK cells in peripheral blood of healthy controls (N = 50), asthmatic subjects (N = 56) and asthma patients with AURVIs (N = 49) were measured by flow cytometry. Gating strategy for analysis of CD3^−^CD56^+^CD16^+^ NK cells population. (**a**) Whole blood cell plot. (**b**) Lymphocytes were gated from whole blood pattern. (**c**) CD3 negative population was gated for NK cells analysis. (**d**) The populations of CD56^+^ CD16^+^ NK cells (Q2) were gated. (**e**) Time trends of peripheral CD3^−^CD56^+^CD16^+^ NK cells in asthma patients with AURVIs on day 1, day 3, day 8 and day 14. *p < 0.05, ** p < 0.01,*** p < 0.001

The proportions of CD3^-^CD56^+^CD16^+^ NK cells were quantitated in peripheral blood of all subjects using FACS Caliber Flow cytometer, as shown in Fig. 2. Compared with healthy controls, the asthmatics had lower proportion of CD3^-^CD56^+^CD16^+^ NK cells at baseline. There was no significant difference in CD3^-^CD56^+^CD16^+^ NK cells frequencies between the two asthma groups. We then focused on the changes of NK cells at different points in time under viral infection setting. The flow cytometry analysis displayed that CD3^-^CD56^+^CD16^+^ NK cells numbers markedly increased up to a maximum [17.64 (7.64 ~ 29.36)%] on day 3 in asthma patients upon respiratory virus infections. The percentages of CD3^-^CD56^+^CD16^+^ NK cells in asthma with AURVIs group decreased to 13.20 (4.62 ~ 21.23) % on day 8 (p < 0.001) and 9.35 (4.32 ~ 18.76) % on day 14 (p < 0.001), respectively, but the numbers were still higher than those at baseline.

### The relationship of CD3^-^CD56^+^CD16^+^NK cells with Th1/Th2 immune response in airways of asthmatics

**Fig. 3:**
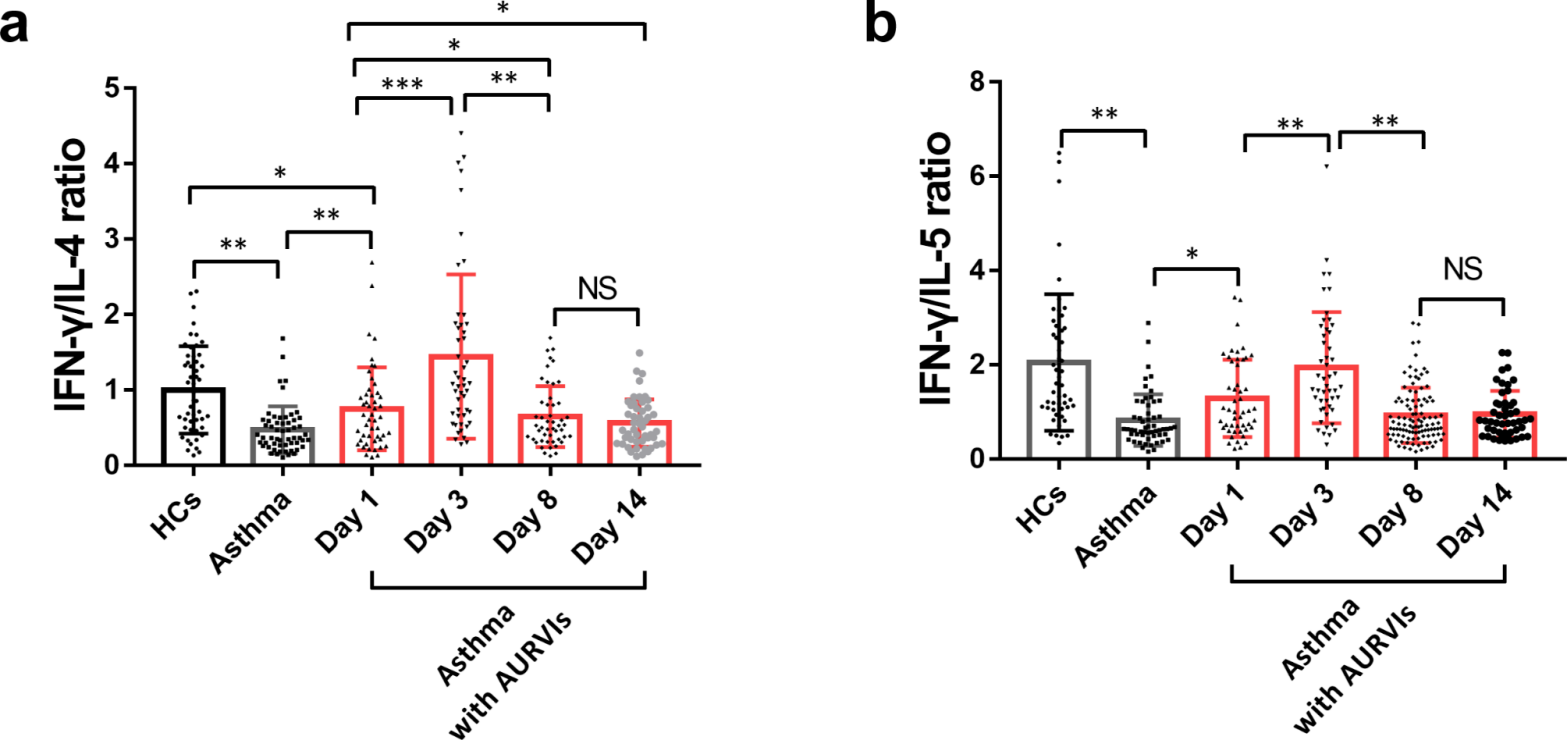
The changes of IFN-γ/IL-4 ratio (**a**) and IFN-γ/IL-5 ratio (**b**) among healthy controls (N = 50), asthmatic subjects (N = 56) and asthma patients with AURVIs (N = 49) on day 1, day 3, day 8 and day 14. *p < 0.05, **p < 0.01, *** p < 0.001

**Fig. 4:**
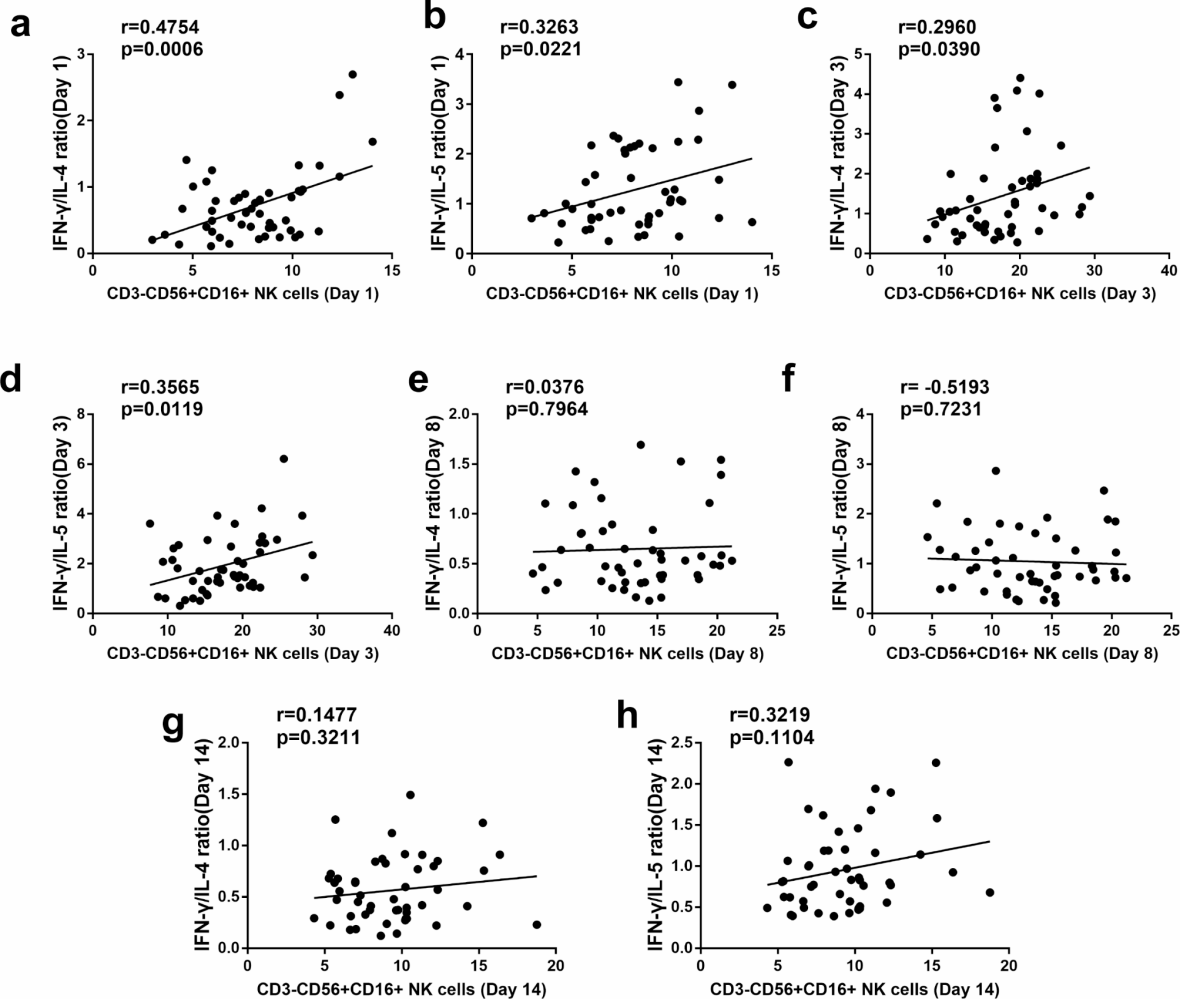
The correlations of peripheral CD56 + CD16 + NK cells populations and IFN-γ/IL-4 ratio (**a**, **c**, **e**, **g**) as well as IFN-γ/IL-5 ratio (**b**, **d**, **f**, **h**) in asthma patients with AURVIs on day 1, day 3, day 8 and day 14

To further investigate the Th1/Th2 balance in airways of asthmatics during viral infection, we analyzed the ratios of IFN-γ/IL-4 and IFN-γ/IL-5 cytokines. As illustrated in Fig. 3, compared with healthy controls, the baseline ratios of IFN-γ/IL-4 (1.00 ± 0.59 versus 0.47 ± 0.31,p < 0.001; 1.00 ± 0.59 versus 0.75 ± 0.55, p < 0.001) and IFN-γ/IL-5 (2.05 ± 1.45 versus 0.83 ± 0.55, p < 0.001; 2.05 ± 1.45 versus 1.29 ± 0.82, p < 0.001) were significantly lower in the two asthma groups. The IFN-γ/IL-4 and IFN-γ/IL-5 ratios of the asthma patients with AURVIs on day 1 were remarkably higher than those in asthma alone group. The data showed that IFN-γ/IL-4 ratios on day 3 were sharply elevated compared with day 1. Then the IFN-γ/IL-4 ratios were detected to decrease on day 8 and maintained until day 14. A similar trend was detected for the changes of the IFN-γ/IL-5 ratios from day 1 to day 14 during the observation period. The linkage between CD3^-^CD56^+^CD16^+^ NK cells and Th1/Th2 immune profile was further analyzed in the asthma patients with AURVIs. The peripheral CD3^-^CD56^+^CD16^+^ NK cells frequencies were positively associated with the IFN-γ/IL-4 ratios on day 1 and day 3 in the phase of respiratory viral infections. Additionally, it was also observed that the proportions of CD3^-^CD56^+^CD16^+^ NK cells in asthma patients with AURVIs had a positive correlation with the IFN-γ/IL-5 ratios on day 1 and day 3, respectively. No relationship was found between CD3^-^CD56^+^CD16^+^ NK cells and Th1/Th2 cytokines ratios on day 8 and day 14 (Fig. 4).

## Discussion

Acute upper respiratory viral infection is a common self-limiting disease, and also an important cause for asthma exacerbation. Viral infection-triggered immune response crucially implicates in the pathogenesis of asthma and asthma exacerbation. Since NK cells are thought to be required for defending against virus, the interaction between NK cells and immune disorder could be possible mechanisms of asthmatic airway inflammation upon virus irritation. Here, we characterized the time trend of Th1/Th2 immunity following acute upper respiratory viral infections in airways of asthma patients, and found a predominant Th1 immune profile in acute phase of upper airway viral infections. We also revealed the upregulated populations of peripheral CD3^−^CD56^+^CD16^+^ NK cells in asthma patients following upper airway viral infections were preferentially related with Th1 immunity. Our results indicated that CD3^−^CD56^+^CD16^+^ NK cells might take an important part in biased immunity of airways in asthma after upper respiratory viral infection.

Respiratory viral infections, including RSV, influenza virus, influenza virus, and parainfluenza virus infections, are greatly associated with asthma exacerbation that subsequently leads to wheezing and reduced expiratory flow rate [16, 17]. Similarly, our study indicated a high incidence of RSV infection among asthma patients with acute upper respiratory viral infection. Also, the asthma subjects with acute viral infection had worsened lung functions as evidenced by the decrease in FEV1. Moreover, our study demonstrated the elevated levels of induced sputum IL-4, IL-5 as well as IFN-γ in response to upper respiratory viral infection, suggesting that viruses exacerbated airway inflammation regardless of their location in respiratory tract. Of note, this study revealed unsynchronized changes with respect to Th1 and Th2 cytokines. Upon viral infections, the IFN-γ levels sharply reached the peak on day 3, while the levels of IL-4 and IL-5 gradually increased up to maximum on day 8. It implied that IFN-γ might principally contribute to the biased immunity toward Th1-like response during acute phase in asthma with viral infection.

NK cells belong to lymphoid cells implicated in the innate and adaptive immunity. Human peripheral blood NK cells can be divided into two populations based on the relative densities of the surface marker CD56 and CD16 in the absence of CD3. CD56^+^CD16^−^(CD56^bright^) subsets are critical for cytokine production with weak cytotoxicity [18–21]. It was known that CD56^+^CD16^+^ (CD56^dim^) subsets, which represent for the major population (more than 90%) of circulating NK cells, possess a high cytolytic capacity against various pathogens and generate low levels of cytokines [22–24]. But other findings reported that CD56^dim^ NK cells act as an important source of cytokines (such as IFN-γ and TNF-α) in innate immune responses [25, 26]. Although NK cells are widely believed to be involved in bacteria and virus defense, it is still controversial on the effect of circulating CD3^−^CD56^+^CD16^+^ NK subsets under asthmatic environments. More importantly, the role of CD3^−^CD56^+^CD16^+^ NK cells in regulating immune response in viral infection-irritated asthma remains elusive. The CD3^−^CD56^+^CD16^+/−^ NK cell subpopulations were previously found to be reduced in allergic diseases including asthma and rhinitis [27], yet some studies exhibited the augmented cytotoxic activities of NK cells in peripheral blood from asthmatic subjects [13]. In this study, we detected the populations of peripheral CD3^−^CD56^+^CD16^+^ NK cells between healthy individuals and asthma subjects. Our results showed that healthy individuals had higher numbers of CD3-CD56 + CD16 + NK cells in peripheral blood compared with asthmatics. It was of interest to note that the asthma patients the phase of acute virus infection had elevated CD3^−^CD56^+^CD16^+^ NK cell numbers than those with asthma alone. These data suggested that CD3^−^CD56^+^CD16^+^ NK cells might be the required participant to counteract viral infection in asthma. Notably, a previous research from Duvall et al. suggested that NK cells displayed disabled functions with reduced numbers under asthmatic conditions [28]. For that reason, we speculated that NK cells activation and functions were positively affected by respiratory viral infections in asthma exacerbations.

NK cells are rapidly responsive innate lymphocytes that can trigger subsequent adaptive immunity in virus-induced asthma exacerbations [9, 29]. Therefore, the present study further determined the possible impact of CD3^−^CD56^+^CD16^+^ NK cells on the basic immunology of asthma subsequent to virus challenges. Following upper respiratory viral infections, the levels of induced sputum IFN-γ in asthma patients were increased prior to Th2 cytokines IL-4 and IL-5. Moreover, we also found that the frequencies of peripheral CD3^−^CD56^+^CD16^+^ NK cells were rapidly augmented on day 1 to day 3, which was positively associated with ratios of IFN-γ/IL-4 and IFN-γ/IL-5. The observations of this study indicated that peripheral CD3^−^CD56^+^CD16^+^ NK cells activation were tightly in correlation with higher IFN-γ expression of asthmatics with AURVIs. Two points might account for this issue. As aforementioned, CD3^−^CD56^+^CD16^+^ NK cells are highly cytotoxic and robust producers of proinflammatory cytokines, thereby leading to dramatically increased IFN-γ production during antiviral responses [21]. For another, virally activated NK cells mediated low-Th2 immunity through inducing innate and adaptive immune cells including airway epithelial cells, neutrophils and Th1 cells that secret type-1 cytokines (e.g. IFN-γ, TNF-α) [30]. Our study suggested the requisite role of CD3^−^CD56^+^CD16^+^ NK cells in low-Th2 immune response, which represented the main phenotype of asthma with acute viral infections. It was noteworthy that the asthmatics with respiratory viral infections showed delayed but lasting Th2 airway response, as evidenced by accumulated upregulation of IL-4 and IL-5 levels until day 14 upon viral infections. Clinically, some asthma patients after AURVIs indeed have cough symptom for more than 2 weeks [31]. This implied that the subsequent Th2 immunity of airways post AURVIs might be a potential risk for uncontrolled asthma.

In summary, we demonstrated an upregulated proportion of peripheral CD3^−^CD56^+^CD16^+^ NK cells in asthma patients following viral upper respiratory infections. Besides, we also found increased CD3^−^CD56^+^CD16^+^ NK cells were positively correlated with predominant Th1-like immune response during the acute phase of respiratory viral infections. The anti-viral Th1-immunity by targeting NK cells may be a possible therapeutic option for infection-induced asthma exacerbation.

## Methods

### Subject

A total of 56 well-controlled mild asthma patients without any viral infections and 49 mild asthma patients with AURVIs, from 18 to 60 years old, were enrolled in this study. A separate group of 50 aged- and gender-matched healthy volunteers who had no past history of asthma or other respiratory diseases from physical examination center of Shanghai East Hospital were recruited as healthy controls. The diagnosis of mild asthma was based on the Global Initiative for Asthma (GINA) guidelines. Acute upper respiratory virus infections were diagnosed as followed: (1) symptoms and signs of nasopharyngitis such as fever, nasal congestion and edema, runny nose, sore throat, fatigue, and general pain; (2) abnormal haemogram manifested as WBC count ≤ 10.0 × 10^9^/l or neutrophils ≤ 80%; (3) normal chest X-ray or only with increased lung texture. Systemic infections, individuals diagnosed with severe cardiopulmonary diseases or kidney failure, pregnant or lactating woman were excluded. PCR virus tests (Health Biomed, Ningbo, China) were performed for detection virus from nasopharyngeal swabs. The study was approved by the Ethics Committee of Shanghai East Hospital affiliated to Tongji University. Written informed consent was obtained from all participants.

### Study design

Patients were asked to attend respiratory outpatient clinic as soon as they had symptoms of AURVIs. All subjects underwent pulmonary function tests, exhaled nitric oxide fraction measurement, examination of induced sputum and peripheral blood before the study. Asthma patients with AURVIs were followed with the collection of induced sputum and peripheral blood samples on days 1, 3, 8 and 14 of clinic visits. Symptoms scores of asthma patients were measured by questionnaires related to asthma including asthma control test (ACT) and asthma control questionnaire (ACQ). Allergic status was assessed by skin prick test (SPT).

### Lung function tests and exhaled nitric oxide fraction measurement

Forced expiratory volume in first second (FEV1) and forced vital capacity (FVC) were measured by a computerized spirometer (Master Screen Body, Jaeger, Germany) according to America Thoracic Society (ATS) standard [32]. Measurement of exhaled nitric oxide fraction (FeNO) was conducted with a NO-measuring device (NIOX MINO) in accordance with the standardized criteria of ATS/ESR recommendation [33].

### Sputum induction and processing

Sputum was induced with an aerosol of 3% hypertonic saline solution using an ultrasonic nebulizer. The thick parts of the induced sputum were selected for further processing. The samples were mixed with a vortex mixer and incubated in a water bath with 0.1% dithionthreitol at 37℃ for 15 min to ensure complete homogenization, and then filtered through nylon gauze (300 meshes). Filtrates were centrifuged at 750×g at room temperature. Cell supernatants were stored at -80℃ for cytokine detection. Centrifugation sediment smears were stained with Wright Giemsa dye and observed under microscope to determine the differential cells counts. A qualified sputum sample was defined as containing less than 10% squamous cells.

### Analysis of inflammatory cytokines in induced sputum

The levels of IgE, Th1-type (IFN-γ) and Th2-type (IL-4 and IL-5) cytokines in sputum supernatants from participants were assessed using a solid-phase sandwich enzyme-linked immunosorbent assay (ELISA) (PeproTech, NJ, USA).

### Flow cytometry assay and gating strategy

4 mL peripheral whole blood from each subject was stored in heparinized vacuum tube and stained with antibodies including Peridinin Chlorophyll Protein Complex (PerCP)-cy5.5 conjugated anti-CD45 (Clone BC8-SA), Fluorescein isothiocyanate (FITC)-conjugated anti-CD3 (Clone SP34-2), PE (phycoerythrin)-conjugated anti-CD56 (Clone R19-760), and FITC-conjugated anti-CD16 (Clone 3G8) at room temperature, according to the manufacturer’s protocol. Then the stained samples were fixed with 1 mL 1×hemolysin in FACS staining buffer for 15 min. To quantitate the NK cells, forward- and side- scattered plots were utilized to gate the lymphocytes (anti-CD45-positive) in the whole blood population. The CD3 negative population was further sorted following the first gate. Then the frequencies of NK cells with anti-CD16-positive and anti-CD56-positive phenotype were identified within the CD3 negative group. The detection of the samples was performed on FACS flow cytometry and the flow data were analyzed using FlowJo software.

### Statistical analysis

Statistical analysis was performed using SPSS 19.0 (SPSS Inc., Chicago, USA) and Graphpad prism 7.0 software (GraphPad Software Inc, La Jolla, USA). Data were expressed as mean ± standard deviation or median. Comparisons within groups were measured by analysis of variance (ANOVA), Kruskal–Wallis test, the Student’s t-test, Pearson chi-square χ2 test, or Fisher’s exact test, as appropriate. Correlations were assessed using Spearman rank correlation analysis. P value < 0.05 was considered statistically significant.

## Data Availability

The data of the present study are available from the corresponding author on reasonable request.

## References

[1] Sockrider M, Fussner L. What Is asthma? Am J Respir Crit Care Med. 2020;202(9):25–6. 10.1164/rccm.2029P25 33124914

[2] Papi A, Brightling C, Pedersen SE, Reddel HK, Asthma (2018). Lancet.

[3] Global Asthma Network. The Global Asthma Report 2014. *www.globalasthmareport.org* (accessed 14. December 2017).

[4] Asher MI, García-Marcos L, Pearce NE, Strachan DP. Trends in worldwide asthma prevalence. Eur Respir J. 2020;56:2002094. 10.1183/13993003.02094-2020 32972987

[5] Jartti T, Bonnelykke K, Elenius V, Feleszko W (2020). Role of viruses in Asthma. Semin Immunopathol.

[6] Castillo JR, Peters SP, Busse WW (2017). Asthma exacerbations: Pathogenesis, Prevention, and treatment. J Allergy Clin Immunol Pract.

[7] Smith DK (2017). Respiratory syncytial virus bronchiolitis in children. Am Fam Physician.

[8] Bjorkstrom NK, Strunz B, Ljunggren HG (2022). Natural killer cells in antiviral immunity. Nat Rev Immunol.

[9] Mujal AMDR, Sun JC (2021). Natural killer cells: from innate to adaptive features. Annual Reviews.

[10] Welsh RM, Waggoner, Stephen N (2013). NK cells controlling virus-specific T cells: rheostats for acute vs. persistent infections. Virology.

[11] Cook KDWS, Whitmire JK (2014). NK cells and their ability to modulate T cells during virus infections. Crit Rev Immunol.

[12] Kucuksezer UC, Aktas Cetin E, Esen F (2021). The role of natural killer cells in autoimmune diseases. Front Immunol.

[13] Long X, Xie J, Zhao K (2016). NK cells contribute to persistent airway inflammation and AHR during the later stage of RSV infection in mice. Med Microbiol Immunol.

[14] Scharenberg MVS, Kekäläinen E (2019). Bergman P,Ameri MA,Johansson N, Sondén K, Jones SF, Färnert A, Ljunggren HG, Michaëlsson J, Sörensen AS, Marquard N. Influenza a virus infection induces hyperresponsiveness in human lung tissue-resident and peripheral blood NK cells. Front Immunol.

[15] Marquardt NKE, Chen P, Kvedaraite E, Wilson JN, Ivarsson MA, Mjösberg J, Berglin L, ML JS (2017). Human lung natural killer cells are predominantly comprised of highly differentiated hypofunctional CD69 – CD56 dim cells. J Allergy Clin Immunol.

[16] Hayashi Y, Sada M, Shirai T, Okayama K, Kimura R, Kondo M, Okodo M, Tsugawa T, Ryo A, Kimura H. Rhinovirus infection and virus-induced asthma. Viruses. 2022;14(12):2616. 10.3390/v14122616 PMC9781665 36560620

[17] Nakagome K, Nagata M (2022). Innate Immune responses by respiratory viruses, including Rhinovirus, during Asthma Exacerbation. Front Immunol.

[18] Cichocki F, Grzywacz B, Miller JS (2019). Human NK Cell Development: one road or many?. Front Immunol.

[19] Poli A, Michel T, Theresine M, Andres E, Hentges F, Zimmer J (2009). CD56bright natural killer (NK) cells: an important NK cell subset. Immunology.

[20] Korsgren M. NK cells and asthma. Curr Pharm Des. 2002;8(20):1871–6. 10.2174/1381612023393738 12171539

[21] Gorska MM (2017). Natural killer cells in asthma. Curr Opin Allergy Clin Immunol.

[22] Cooper MA, Fehniger TA (2001). The biology of human natural killer-cell subsets. Trends Immunol.

[23] Sheppard S, Sun JC. Virus-specific NK cell memory. J Exp Med. 2021;218(4). 10.1084/jem.20201731 PMC7992500 33755720

[24] Busse WWLRJ, Gern JE (2010). Role of viral respiratory infections in asthma and asthma exacerbation. Lancet.

[25] Mariaa ADBF, Cantonic C, Moretta L (2011). Revisiting human natural killer cell subset function revealed cytolytic CD56dimCD16 + NK cells as rapid producers of abundant IFN-γ on activation. PNAS.

[26] Fauriat CLE, Ljunggren HG, Bryceson YT (2010). Regulation of human NK-cell cytokine and chemokine production by target cell. Blood.

[27] Deniz G, van de Veen W, Akdis M (2013). Natural killer cells in patients with allergic Diseases. J Allergy Clin Immunol.

[28] Duvall MG, Barnig C, Cernadas M (2017). Natural killer cell-mediated inflammation resolution is disabled in severe asthma. Sci Immunol.

[29] Lepretre FGD, Chanez P, Duez C (2023). Natural killer cells in the lung potential role in asthma and virus-induced excerbation. Eur RESPIRATORY Rev.

[30] Camiolo MJ, Kale SL, Oriss TB, Gauthier M, Ray A (2021). Immune responses and exacerbations in severe Asthma. Curr Opin Immunol.

[31] Eccles R, Dicpinigaitis P, Turner RB, Druce HM, Adeleke M, Mann AL (2016). Characterization of urge to cough and cough symptoms associated with the Common Cold: results of a US internet survey. Postgrad Med.

[32] Miller MR, Hankinson J, Brusasco V (2005). Standardisation of spirometry. Eur Respir J.

[33] Reddel HK, Taylor DR, Bateman ED (2009). An official American Thoracic Society/European Respiratory Society statement: asthma control and exacerbations: standardizing endpoints for clinical asthma trials and clinical practice. Am J Respir Crit Care Med.

